# Tackling Multiple Biologic Failure in Psoriasis: Research and Advocacy Perspectives

**DOI:** 10.1177/24755303241288536

**Published:** 2024-09-30

**Authors:** Kathryn Haran, George Gondo, Payton Smith, Chandler Johnson, Allison Kranyak, Tina Bhutani, Wilson Liao

**Affiliations:** 1 Department of Dermatology, 8785 University of California San Francisco, San Francisco, CA, USA; 2 427145 National Psoriasis Foundation, Portland, OR, USA

**Keywords:** psoriasis, biologic, treatment, psoriasis, advocacy, registry, treatment outcomes, treatment, psoriasis

## Abstract

Psoriasis is an immune-mediated skin disease commonly treated with biologic therapies. While there are currently 12 different biologics approved for psoriasis, there still exists a challenging subset of patients who have tried and failed biologics from multiple classes. In this commentary, we discuss the research and advocacy-based efforts by the National Psoriasis Foundation (NPF) and academic collaborators to understand and better support multiple biologic failure (MBF) psoriasis patients. The NPF MBF registry will gather clinical and demographic information on MBF patients to improve therapeutic outcomes, while legislative efforts through NPF Capitol Hill Day aim to advance federal laws in support of all psoriasis patients, with a recent focus on access to biologic therapy. Together, these 2 efforts will improve the care for all people living with psoriasis, including those experiencing MBF.

## Commentary

Psoriasis is an inflammatory skin disease affecting 125 million people worldwide.^1^ Frequently prescribed treatments include topicals, phototherapy, and biologics.^1^ Despite the increasing number and types of biologics, a population of patients with psoriasis exists who have tried and failed multiple biologic therapies, posing a significant challenge.^2^ While the cause of multiple biologic failure (MBF) is multifaceted, possible contributors include the development of anti-drug antibodies and genetic predisposition.^3,4^ This commentary describes the National Psoriasis Foundation (NPF) MBF registry and other unique advocacy-based opportunities to improve care for patients with psoriasis receiving biologics.

Recent research efforts have focused on using large psoriasis databases to characterize the sociodemographic and clinical characteristics of MBF patients. One recent study by our group using the CorEvitas psoriasis registry found that female sex and hyperlipemia were associated with MBF.^2^ However, there is no MBF-specific data collection tool or registry.^2^ To address this need, the National Psoriasis Foundation (NPF) has developed a cloud-based registry that crowdsources clinical data from dermatologists and patients to gather data on MBF patients and compares them to a control group of biologic good response patients.

The NPF MBF registry is recruiting patients at academic centers across the United States, with the goal of enrolling at least 1000 patients. Participants are individuals 18 years of age or older who have a diagnosis of moderate to severe plaque psoriasis. The MBF group includes patients who have tried at least 3 biologics from a minimum of 2 mechanistic classes (TNF, IL-17, IL12/23, IL-23 inhibitors) for at least 3 months each, while the control group includes patients who have only ever been on a single biologic for at least 2 years. Both study groups complete questionnaires about their psoriasis, demographics, and comorbid conditions. Providers complete a questionnaire for each patient enrolled, which includes clinical assessments such as the physician’s global assessment and a review of all psoriasis therapies tried by the patient. Ultimately, analyzing data from this registry will advance our understanding of the factors associated with multiple biologic failure in psoriasis patients, to improve treatment selection and therapeutic outcomes.

While the NPF MBF registry will gather data to fill gaps in our understanding of psoriasis multiple biologic failure, it is important to recognize that this endeavor will take time. A more immediate way to help patients, including MBF patients, is through advocacy. Several co-authors had the privilege of participating in the National Psoriasis Foundation Capitol Hill Day, where patients living with psoriasis and psoriatic arthritis, physicians, researchers, and NPF staff collaborated to advocate for legislative change related to psoriasis.^5^ This included advocating for bills like the Safe Step Act, which mandates patient-centered exceptions to insurance step-therapy protocols, such as not requiring patients to try a biologic therapy that is medically contraindicated.^6^ The NPF has successfully advocated for similar legislation at the state level.

The combined efforts of scientific research and advocacy for legislative change will ultimately advance psoriasis care. The NPF is helping lead that charge via its MBF registry and Capitol Hill Day initiatives.
